# Selective disruption of synaptic NMDA receptors of the hippocampal trisynaptic circuit in Aβ pathology

**DOI:** 10.1186/s40659-024-00537-7

**Published:** 2024-08-22

**Authors:** Rocio Alfaro-Ruiz, Alejandro Martín-Belmonte, Carolina Aguado, Ana Esther Moreno-Martínez, Yugo Fukazawa, Rafael Luján

**Affiliations:** 1 Synaptic Structure Laboratory, Departamento de Ciencias Médicas, Facultad de Medicina, Instituto de Biomedicina de la UCLM (IB-UCLM), Universidad Castilla-La Mancha, Campus Biosanitario, C/ Almansa 14, Albacete, 02008 Spain; 2 Laboratorio de Estructura Sináptica, Instituto de Investigación Sanitaria de Castilla-La Mancha (IDISCAM), Albacete, Spain; 3 Pharmacology Unit, Department of Pathology and Experimental Therapeutics, Faculty of Medicine and Health Sciences, Institute of Neurosciences, University of Barcelona, L’Hospitalet de Llobregat, 08907 Spain; 4 Neuropharmacology and Pain Group, Neuroscience Program, Institut d’Investigació Biomèdica de Bellvitge, IDIBELL, L’Hospitalet de Llobregat, 08907 Spain; 5 Division of Brain Structure and Function, Faculty of Medical Science, University of Fukui, Fukui, Japan; 6 Life Science Innovation Center, University of Fukui, Fukui, Japan

**Keywords:** Alzheimer´s disease, Hippocampus, NMDA receptors, Immunohistochemistry, Electron microscopy, Freeze-fracture, AD mouse model

## Abstract

Synaptic dysfunction is an early feature in Alzheimer’s disease (AD) pathogenesis and a major morphological correlate of memory deficits. Given the main synaptic location of *N*-methyl-D-aspartate receptors (NMDARs), their dysregulation has been implicated in these pathological effects. Here, to detect possible alterations in the expression and synaptic localisation of the GluN1 subunit in the brain of amyloidogenic APP/PS1 mice, we employed histoblot and SDS-digested freeze-fracture replica labelling (SDS-FRL) techniques. Histoblots showed that GluN1 expression was significantly reduced in the hippocampus in a layer-dependent manner, in the cortex and the caudate putamen of APP/PS1 transgenic mice at 12 months of age but was unaltered at 1 and 6 months. Using quantitative SDS-FRL, we unravelled the molecular organisation of GluN1 in seven excitatory synapse populations at a high spatial resolution in the CA1 and CA3 fields and the DG of the hippocampus in 12-month-old APP/PS1 mice. In the CA1 field, the labelling density for GluN1 in the excitatory synapses established on spines and interneurons, was significantly reduced in APP/PS1 mice compared to age-matched wild-type mice in the *stratum lacunosum-moleculare* but unaltered in the *stratum radiatum*. In the CA3 field, synaptic GluN1 was reduced in mossy fibre-CA3 pyramidal cell synapses but unaltered in the A/C-CA3 pyramidal cell synapses. In the DG, the density of GluN1 in granule cell-perforant pathway synapses was reduced in APP/PS1 mice. Altogether, our findings provide evidence of specific alterations of synaptic GluN1 in the trisynaptic circuit of the hippocampus in Aβ pathology. This differential vulnerability in the disruption of NMDARs may be involved in the mechanisms causing abnormal network activity of the hippocampal circuit and cognitive impairment characteristic of APP/PS1 mice.

## Introduction

Glutamate and its receptors, traditionally categorised as ionotropic and metabotropic, are largely responsible for excitatory neurotransmission in the central nervous system [1]. Ionotropic glutamate receptors are further subdivided based on selectivity for agonists, into alpha-amino-3-hydroxy-5-methylisoxazole-4-propionic acid receptor (AMPAR), *N*-methyl-*D*-aspartate receptor (NMDAR), and kainate receptors [1]. The high Ca^2+^ permeability of NMDARs is a key feature which plays a central role in several physiological processes, including neuron differentiation, synapse consolidation in the developing brain and activity-dependent forms of synaptic plasticity [1]. Their excessive activation has been implicated in mechanisms of neuronal death in hypoxia-ischemia, epilepsy, and neurodegenerative disorders such as Alzheimer’s disease (AD) [2–4]. Impairments in memory are considered cognitive hallmarks of AD that can be linked to the neuropathological features of the disease, including the formation of senile plaques of amyloid-β (Aβ), neurofibrillary tangles (NFT) of phospho tau, synapse loss and neuronal loss [5].

To date at least seven subunits of the NMDAR have been identified: one GluN1 subunit, four GluN2 subunits (GluN2A, GluN2B, GluN2C, and GluN2D), and two GluN3 subunits (GluN3A and GluN3B) [1]. Functional NMDARs are assembled as heterotetramers containing two GluN1 obligatory subunits and two regulatory subunits GluN2 or GluN3 which confer different biophysical properties [1, 2, 6]. The GluN1 subunit is expressed ubiquitously in the brain, while the GluN2 subunits show marked regional variations [6, 7], but both are most strongly expressed in the hippocampus [6, 8, 9], a brain region known to be critical for the acquisition of episodic memory [10]. This cognitive process is altered in patients with Mild Cognitive Impairment (MCI), a preclinical phase of AD, that is linked to circuit-specific structural and functional disruptions in the hippocampal CA1 and CA3/dentate regions [11, 12].

Biochemical and anatomical data indicate that NMDARs are enriched in postsynaptic densities (PSDs) of hippocampal excitatory synapses [13–16]. At that location, NMDARs are responsible for mostly fast excitatory transmission in the hippocampus, where they play an essential role in the strengthening of synapses through long-term potentiation (LTP), the cellular mechanism underlying learning and memory, at the CA1 Schaffer collateral synapse [17], the CA3 recurrent associational/commissural synapse (A/C synapse), the mossy fibre (MF)-CA3 synapse [18] and the perforant path–granule cell synapse in the dentate gyrus (DG) [19]. Dysregulation of synaptic plasticity is taking place in AD [20]. Consequently, any alteration in the number and density of NMDARs could contribute to the synaptic and memory deficits that are associated with AD.

Recently, we reported a decreased density of GluN1 in excitatory synapses on spines and interneurons in the hippocampal CA1 field of a tauopathy mouse model [21]. How the molecular organisation of synaptic NMDARs is affected by amyloidosis in the hippocampus has not been explored. Furthermore, it is not known if specific hippocampal synapses of the trisynaptic circuit are differentially affected by Aβ pathology, or if synaptic deficits appear at the same age in APP/PS1 mice. Here, we show that hippocampal synapses are not equally affected by Aβ pathology in the trisynaptic circuit and demonstrate a reduction in synaptic NMDARs at specific excitatory synapses in the CA1 and CA3 subfields and the DG molecular layer in APP/PS1 mice.

## Materials and methods

### Animals

Male APP/PS1 mice (RRID: IMSR_MMRRC:034832) were obtained from the Jackson Laboratory (https://www.jax.org/strain/005864) and expressed Mo/Hu APP695swe construct in conjunction with the exon-9-deleted variant of human presenilin 1 [Tg(APPswe, PSEN1dE9)85Dbo/Mmjax] [22, 23]. The “control” wild type (WT) mice were age-matched littermates without the transgene. The following ages were selected for analysis: (i) no sign of pathology (1 month), used as a preclinical stage (ii) first signs of Aβ deposition (6 months), used as the beginning of AD pathology [23] and (iii) onset of memory deficits with severe synapse loss and widespread Aβ deposition (12 months), used as advanced stage of AD pathology [24, 25]. For each age and genotype, four mice were used for histoblotting and three mice were used for SDS-digested freeze-fracture replica labelling (SDS-FRL). All mice were maintained at the Animal House Facility of the University of Castilla-La Mancha (Albacete, Spain) in cages of 2 or more mice, on a 12-hour light/12-hour dark cycle at 24 °C and received food and water *ad libitum*. Care and handling of animals prior to and during experimental procedures were in accordance with Spanish (RD 53/2013) and European Union regulations (2010/63/UE), and all protocols and methodologies were approved by the local Animal Care and Use Committee.

For histoblotting, animals were deeply anesthetised by intraperitoneal injection of ketamine/xylazine 1:1 (ketamine, 100 mg/Kg; xylazine, 10 mg/Kg), the brain was dissected, frozen rapidly in liquid nitrogen and stored at -80ºC. For SDS-FRL experiments, animals were anesthetised with sodium pentobarbital (50 mg/kg, i.p.) and perfused transcardially with 25 mM PBS for 1 min, followed by perfusion with 2% paraformaldehyde in 0.1 M phosphate buffer (PB) for 12 min. After perfusion, brains were removed, and the hippocampi were dissected and cut into coronal slices (130 μm) using a Microslicer (Dosaka, Kyoto, Japan) in 0.1 M PB.

### Antibodies and chemicals

Mouse monoclonal antibody, raised against the GluN1 subunit of NMDARs was used to detect the protein of interest (MAB363, Millipore Bioscience Research Reagents). This antibody was directed against the extracellular loop of GluN1, and its specificity was characterised previously [26]. The secondary antibodies used were as follows: alkaline phosphatase (AP)-goat anti-mouse IgG (H + L) and anti-mouse IgG conjugated to 10 nm gold particles (1 : 100; British Biocell International, Cardiff, UK).

### Histoblotting

The regional distribution of GluN1 was analysed in mouse brains, using the histoblot technique [27]. Briefly, horizontal cryostat sections (10 μm) from mouse brain were overlapped with nitrocellulose membranes moistened with 48 mM Tris-base, 39 mM glycine, 2% (w ⁄v) sodium dodecyl sulphate and 20% (v ⁄v) methanol for 15 min at room temperature (~ 20 ºC). After blocking in 5% (w ⁄v) non-fat dry milk in phosphate-buffered saline with Tween for 1 h, nitrocellulose membranes were treated with DNase I (5 U ⁄mL), washed and incubated in 2% (w ⁄v) sodium dodecyl sulphate and 100 mM β-mercaptoethanol in 100 mM Tris–HCl (pH 7.0) for 60 min at 45ºC to remove adhering tissue residues. After extensive washing, the blots were incubated in the anti-GluN1 antibody (0.5 mg ⁄mL) in blocking solution overnight at 4ºC. The bound primary antibodies were detected with alkaline phosphatase-conjugated anti-mouse IgG secondary antibodies [27]. A series of primary and secondary antibody dilutions and incubation times were used to optimise the experimental conditions for the linear sensitivity range of the alkaline phosphatase reactions. To compare the expression levels of NMDARs between the wild type and APP/PS1 mice and at all ages, all nitrocellulose membranes were processed in parallel, and the same incubation time for each reagent was used for the antibody. Digital images were acquired by scanning the nitrocellulose membranes using a desktop scanner (HP Scanjet 8300). Image analysis and processing were performed using the Adobe Photoshop software (Adobe Systems, San Jose, CA, USA) as described previously [28].

### SDS-digested freeze-fracture replica labelling

Immunohistochemical reactions at the electron microscopic level were carried out using the SDS-FRL methods as described earlier [29]. Briefly, hippocampal slices were trimmed containing the CA1 field or the CA3 field or the DG and immersed into graded glycerol of 10–30% (v/v) in 0.1 M PB at 4 °C overnight. Slices were frozen using a high-pressure freezing machine (HPM010, BAL-TEC, Balzers, Liechtenstein). Slices were then fractured into two parts at -120 °C and replicated by carbon deposition (5 nm thick), platinum (60º unidirectional from horizontal level, 2 nm), and carbon (15–20 nm) in a freeze-fracture replica machine (BAF060, BAL-TEC, Balzers, Liechtenstein). Replicas were transferred to 2.5% (w/v) SDS and 20% (w/v) sucrose in 15 mM Tris buffer (pH 8.3) for 18 h at 80 °C with shaking to dissolve tissue debris. The replicas were washed three times in 50 mM Tris-buffered saline (TBS, pH 7.4), containing 0.05% (w/v) bovine serum albumin (BSA), and then blocked with 5% (w/v) BSA in the washing buffer for 1 h at room temperature. The replicas were then washed and reacted with a mouse monoclonal antibody against the GluN1 subunit of NMDARs (10 µg /ml), diluted in 25 mM TBS containing 1% (w/v) BSA overnight at 15 °C. Following three washes in 0.05% BSA in TBS and blocking in 5% (w/v) BSA/TBS, replicas were incubated in goat anti-mouse IgGs coupled to 10 nm gold particles (1:30; British Biocell International, Cardiff, UK) diluted in 25 mM TBS containing 5% (w/v) BSA overnight at room temperature. When the primary antibody was omitted, no immunoreactivity was observed. After immunogold labelling, the replicas were immediately rinsed three times with 0.05% BSA in TBS, washed twice with distilled water, and picked up onto grids coated with pioloform (Agar Scientific, Stansted, Essex, UK).

### Quantification and analysis of SDS-FRIL data

The labelled replicas were examined using a transmission electron microscope (JEOL-1400Flash) equipped with a digital high-sensitivity sCMOS camera, and images captured at different magnifications. The antibody used in this study was visualised by immunoparticles on the exoplasmic face (E-face), consistent with the extracellular location of its epitope. Digitised images were then modified for brightness and contrast using Adobe PhotoShop CS5 (Mountain View, CA, USA) to optimise them for quantitative analysis.

*Number and density of GluN1 immunoparticles at synaptic sites.* The GluN1 immunoparticles composing excitatory synapses of spines and shafts of pyramidal cells and interneuron dendrites located in the *strata radiatum* and *lacunosum-moleculare* of the CA1 field, the *stratum lucidum* and *stratum radiatum* of the CA3 field and the outer two thirds of the molecular layer of the DG, in the two genotypes (wild type and APP/PS1), were determined at 12 months of age. For this purpose, the software GPDQ (*Gold Particle Detection and Quantification*) developed to perform automated and semi-automated detection of gold particles present in each neuronal compartment was used [30]. Most of the spines in the CA1 field, CA3 field and DG arise from principal cells, whereas dendritic shafts receiving several synapses are considered to originate from interneurons.

Quantitative analysis of immunogold labelling for GluN1 was performed on excitatory postsynaptic specialisations indicated by the presence of intramembrane particle (IMP) clusters on the exoplasmic face (E-face). Excitatory postsynaptic specialisations were considered as such when IMP clusters contained at least 30 intramembrane particles. One of the advantages of the SDS-FRL technique is that the whole synaptic specialisation of excitatory synapses plasma membrane is immediately visible over the surface of neurons. The outline of postsynaptic specialisation (IMP clusters) was manually demarcated by connecting the outermost IMP particles. The area of synaptic sites was measured using the software GPDQ.

Immunogold particles for GluN1 were regarded as synaptic labelling if they were within demarcated IMP clusters and those located in the immediate vicinity within 30 nm from the edge of the IMP clusters, given the potential distance between the immunogold particles and antigens. The density of the immunoparticles for GluN1 in each synaptic site was calculated by dividing the number of the immunoparticles by the area of the demarcated IMP clusters. Measurements were performed on three animals, and results were pooled because the densities for immunogold particles were not significantly different in those animals. Immunoparticle densities were presented as mean ± SEM between animals.

### Controls

To test method specificity in the procedures for electron microscopy, replicas were incubated according to the protocol described above with primary antibodies omitted or replaced with 1% (v/v) normal goat serum. Labelling densities on clusters of intramembrane particles were < 1.2 particles/µm^2^ in these cases.

### Data analysis

To avoid observer bias, blinded experiments were performed for immunoblots and immunohistochemistry prior to data analysis. Statistical analyses were performed using GraphPad Prism (San Diego, Ca, USA) and data were presented as mean ± SEM unless indicated otherwise. Statistical significance was defined as *p* < 0.05. The statistical evaluation was performed using the Shapiro–Wilk normality test and Kolmogorov–Smirnov test for the study of normal distribution and an unpaired *t*-test for the comparison of variances. If normal distribution or variances were significatively different, the samples were considered as non-parametric and analysed by Mann–Whitney test; otherwise, they were considered parametric and analysed by unpaired *t*-test. Correlations were assessed using Pearson’s correlation test.

## Results

### Age and region-dependent alteration in GluN1 brain expression in APP/PS1 mice

**Fig. 1:**
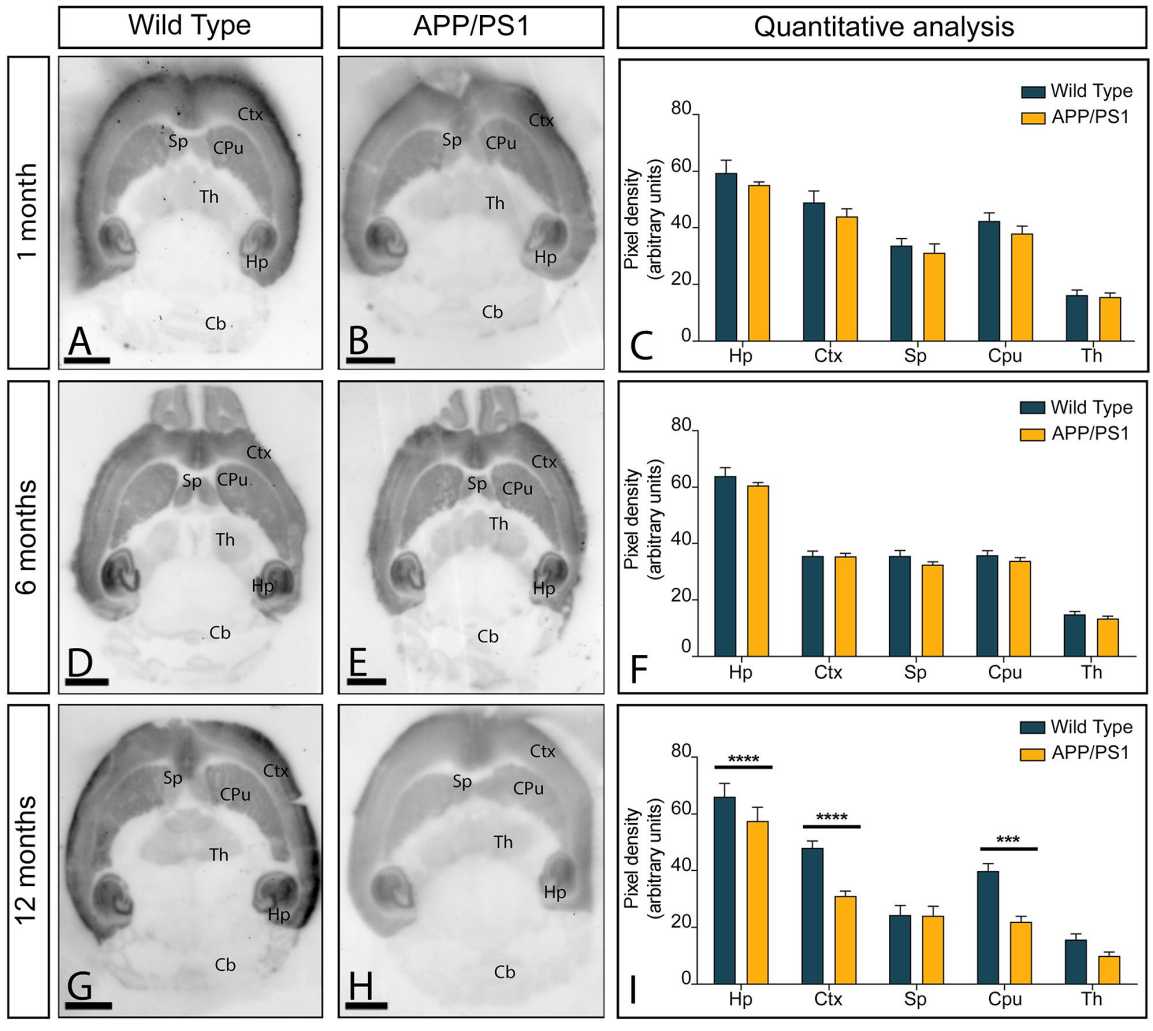
Brain expression of NMDARs in APP/PS1 mice. (**A**-**I**) The expression of the GluN1 protein was visualised using histoblots of horizontal brain sections at 1, 6 and 12 months of age in wild type and APP/PS1 mice using an affinity-purified anti-GluN1 antibody. Densitometric analysis of scanned histoblots allowed determination of GluN1 expression in different brain regions. The strongest GluN1 expression was detected in the hippocampus (Hp), followed by the cortex (Ctx). Moderate expression levels were detected in the caudate putamen (CPu) and the septum (Sp). The weakest expression levels were found in the thalamus (Th), and the cerebellum (Cb). Densitometry data generated at 1 and 6 months of age showed no differences in GluN1 expression in APP/PS1 mice compared to age-matched wild type controls, but a significant reduction was detected in the hippocampus, cortex and caudate putamen at 12 months of age (Mann-Whitney test, **** *P* < 0,0001, *** *P* < 0,001). Error bars indicate SEM. Scale bars: 0.25 cm

The region-dependent alterations in NMDAR expression in the brain of APP/PS1 and age-matched wild type mice were determined using a GluN1 subunit-specific antibody in conventional histoblots [27] at 1, 6 and 12 months of age (Fig. 1A-I). This technique is a reliable way to analyse the brain expression of different proteins without compromising the integrity of antibody-binding sites by tissue fixation that is commonly required for immunohistochemistry [27]. In wild type mice at the three ages, immunolabelling for GluN1 was distributed in the brain, with strong labelling in the hippocampus and the neocortex, followed by the caudate putamen and septum (Fig. 1A, D,G). Moderate labelling was found in the thalamus and weak in the cerebellum and midbrain nuclei (Fig. 1A, D,G). In APP/PS1 mice, this GluN1 expression pattern was quantitatively very similar in APP/PS1 mice at 1 (Fig. 1C), 6 (Fig. 1F), but not at 12 months of age, when a significant decrease in GluN1 labelling was observed in the hippocampus, cortex and caudate putamen (Fig. 1I).

### Layer-dependent alteration in GluN1 hippocampal expression in APP/PS1 mice

**Fig. 2:**
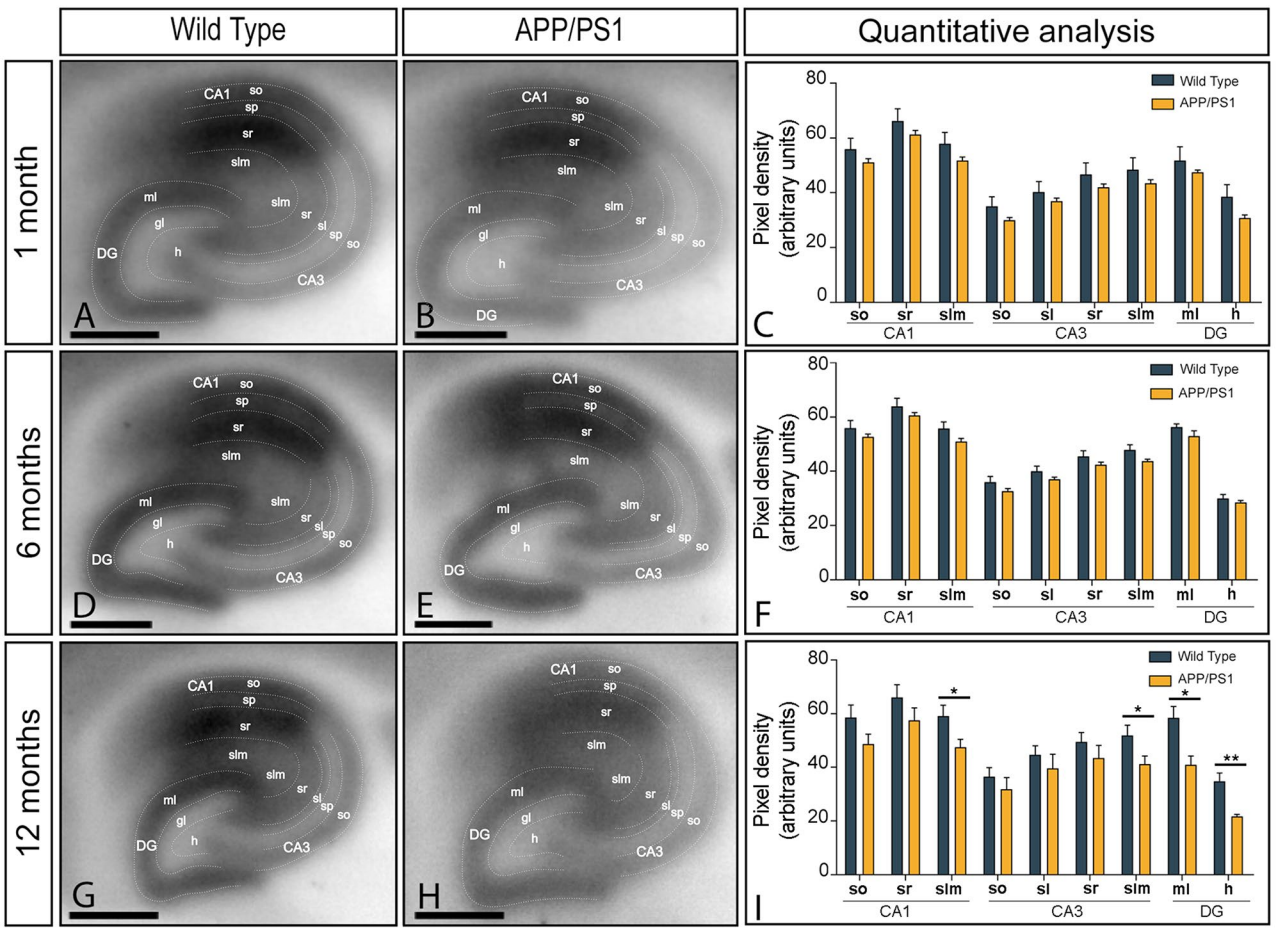
Layer expression of NMDARs in the hippocampus of APP/PS1 mice. (**A**-**I**) The hippocampal layer expression of the GluN1 protein was visualised using histoblots of horizontal brain sections at 1, 6 and 12 months of age in wild type and APP/PS1 mice using an affinity-purified anti-GluN1 antibody. Densitometric analysis of scanned histoblots allowed determination of GluN1 expression in different hippocampal subfields and dendritic layers, delineated with faint dotted lines. The expression of GluN1 was strong in all dendritic layers of the CA1 and CA3 fields and DG. Densitometry data generated at 1 and 6 months of age showed no differences in GluN1 expression in APP/PS1 mice compared to age-matched wild type controls. However, the expression of GluN1 was significantly reduced in the *stratum lacunosum-moleculare* of the CA1 and CA3 fields and the molecular layer and hilus of the DG of APP/PS1 mice at 12 months of age (Mann-Whitney test, * *P* < 0,05; ** *P* < 0,01). Error bars indicate SEM. *Abbreviations*: CA1 field of the hippocampus; CA3, CA3 field of the hippocampus; DG, dentate gyrus; so, *stratum oriens*; sp, *stratum pyramidale*; sr, *stratum radiatum*; slm, *stratum lacunosum-moleculare*; ml, molecular layer; gc, granule cell layer; h, hilus. Scale bars: 0.05 cm

The layer expression pattern of GluN1 in the hippocampus was explored using the histoblot technique (Fig. 2A-I). GluN1 was strongly expressed in all hippocampal subfields and dendritic layers at the three ages of wild type and APP/PS1 mice (Fig. 2A-I). In the CA1 field of wild type and APP/PS1 mice, GluN1 expression was strong in the *strata oriens* (so), *radiatum* (sr) and *lacunosum-moleculare* (slm) at 1, 6 and 12 months of age (Fig. 2A-I). The expression levels of GluN1 were moderate in the so, *stratum lucidum* (sl), sr and slm of the CA3 field (Fig. 2A-I). In the DG, GluN1 expression was strong in the molecular layer and moderate in the hilus (Fig. 2A-I). The quantitative analysis of immunolabelling performed at the three ages indicated that the layer labelling pattern was unchanged in wild type and APP/PS1 mice at 1 and 6 months of age (Fig. 2C, F). However, the expression of GluN1 was significantly reduced in the slm of the CA1 and CA3 fields, and the molecular layer and hilus of the DG of APP/PS1 mice, compared to age-matched wild type controls mice, at 12 months of age (Fig. 2I).

### Altered number and density of NMDARs at CA1 synapses in APP/PS1 mice

We utilised the SDS-FRL technique to determine the number and density of GluN1 in seven populations of excitatory synapses of the hippocampal trisynaptic circuit at 12 months of age: (i) CA1 pyramidal cell synapses in the SR; (ii) CA1 interneuron synapses in the SR; (iii) CA1 pyramidal cell synapses in the SLM; (iv) CA1 interneuron synapses in the SLM; (v) CA3 pyramidal cells-mossy fibre (PC-MF) synapses, (vi) CA3 pyramidal cells-associational/commissural (A/C) synapses, and (vii) DG perforant path synapses. The content of GluN1 synapses varied between synapse types, as described below.

**Fig. 3:**
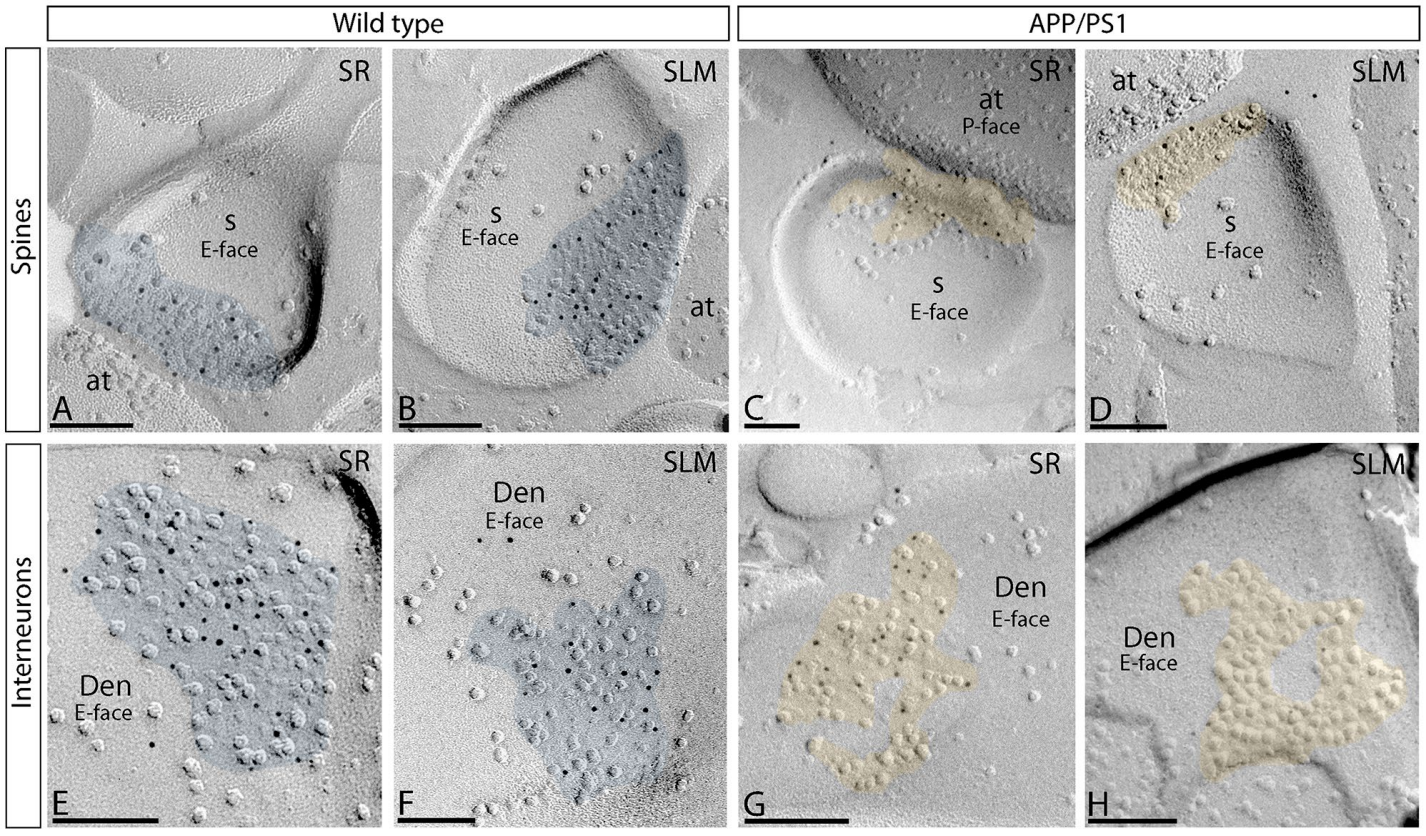
Reduced density of synaptic NMDARs in CA1 neurons of APP/PS1 mice. (**A**-**D**) Electron micrographs of the hippocampus showing immunoparticles for GluN1 at excitatory synaptic sites of pyramidal cell spines (panels **A** to **D**) and interneurons (panels **E** to **H**) in two dendritic layers of the CA1 field, as detected using the SDS-FRL technique in wild type and APP/PS1 mice at 12 months of age. Postsynaptic membrane specialisations (coloured with transparency in blue for wild type and in yellow for APP/PS1) in both spines and interneurons show strong immunoreactivity for GluN1 in wild type in *strata radiatum* and *lacunosum-moleculare*, but weak immunoreactivity only in the *stratum lacunosum-moleculare* of APP/PS1 mice. Scale bars: **A**-**D**, 100 nm; **E**-**H**, 200 nm

The analysis of excitatory synapses on pyramidal cell spines and interneurons was carried out firstly in the SR and SLM of the CA1 field. In wild type mice, immunoparticles for GluN1 in spines were found almost exclusively on IMP clusters regarded as postsynaptic membrane specialisation (PSD) on E-face profiles (Fig. 3A, B). Immunoparticles were randomly distributed over the entire surface of PSDs without forming clusters, although labelling density varied from cluster to cluster (Fig. 3A, B). In APP/PS1 mice, a similar distribution pattern was observed on the excitatory synapses of spines in the SR, but fewer GluN1 immunoparticles were detected in the SLM (Fig. 3C, D). The analysis of excitatory synapses on interneurons (Fig. 3E-H) was next carried out. Similar to spines, most GluN1 immunoparticles in excitatory synapses of interneurons were found over the PSD with no apparent clustering in the SR and SLM of the CA1 field (Fig. 3E, F) but detected at a lower frequency in APP/PS1 mice only in the SLM (Fig. 3G, H).

**Fig. 4:**
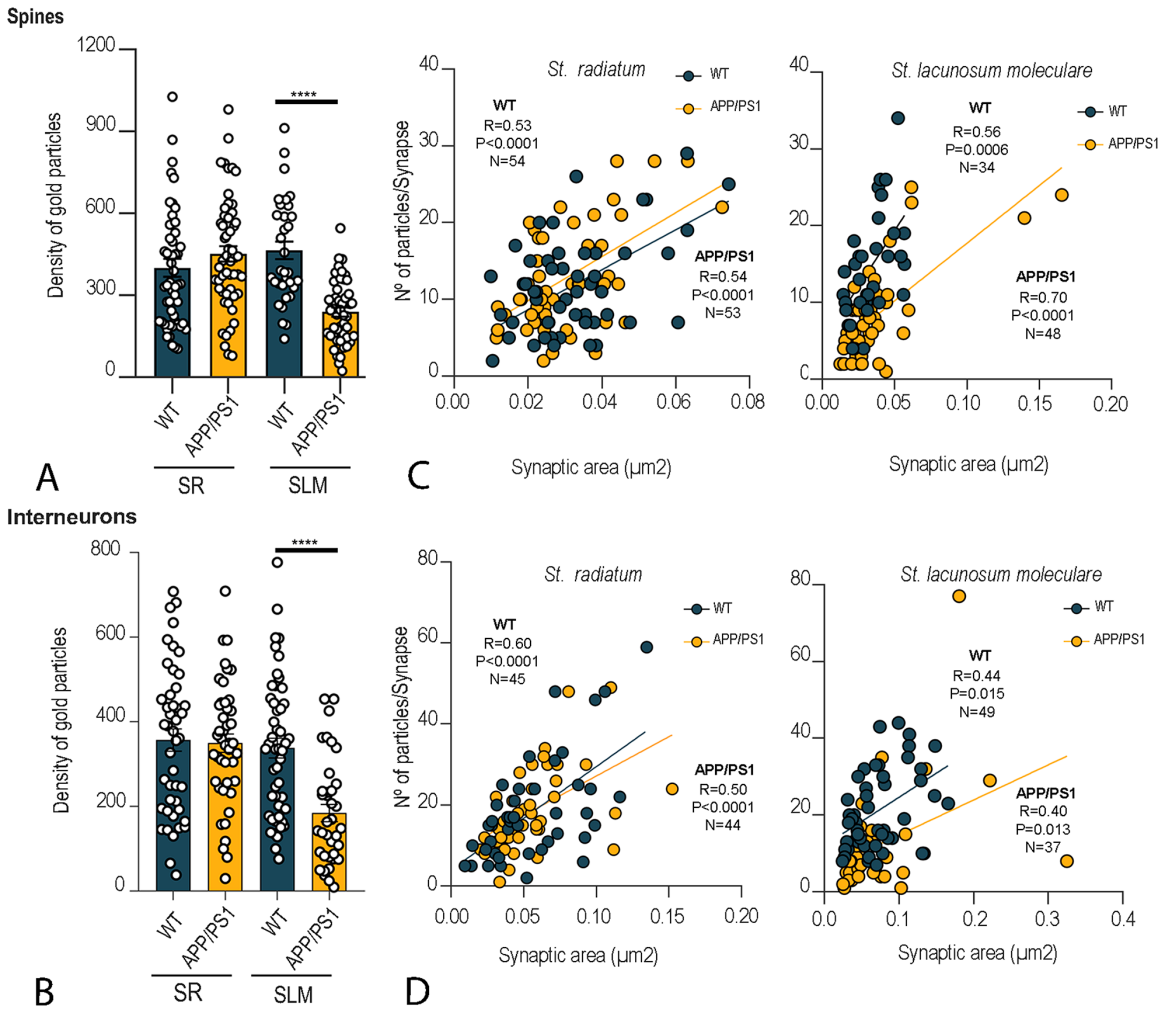
NMDAR immunoparticle density at excitatory synapses on spines and interneurons. (**A**,** B**) Quantitative analysis showing mean densities of GluN1 in excitatory synapses in spines (panel **A**) and interneurons (panel **B**). A significant reduction in the density of immunoparticles for GluN1 was detected in both compartments located in the *stratum lacunosum-moleculare* (SLM) of the CA1 field of APP/PS1 mice (*n* = 3 animals per genotype; Mann-Whitney U test, ****P < 0.0001) compared to age matched wild type. No differences were detected in the *stratum radiatum* (SR) of the CA1 field. (**C**,** D**) Correlation of the number of GluN1 immunoparticles and IMP-cluster area on spines and interneuron dendrites. Scatterplots of the number of immunoparticles for GluN1 versus size of excitatory synapses in both wild type and APP/PS1 mice. In the *strata radiatum* and *lacunosum-moleculare* there is a positive linear correlation between immunoparticle number and synaptic size in both genotypes (Pearson’s correlation test)

The possible differences in the content of synaptic GluN1 between wild type and APP/PS1 mice were tested (Fig. 4; Table 1). A significant reduction in GluN1 levels in excitatory synapses on spines and interneurons was only observed in the SLM in APP/PS1 mice (spines: 237.8 ± 16.6 particles/µm^2^; interneurons: 184.4 ± 20.5 particles/µm^2^) compared to age-matched wild type controls (spines: 463.7 ± 32.7 particles/µm^2^; interneurons: 337.9 ± 23.3 particles/µm^2^) (Mann-Whitney U test, ****P < 0.0001), but not in the SR in APP/PS1 mice (spines: 451.1 ± 28.4 particles/µm^2^; interneurons: 349.60 ± 21.90 particles/µm^2^) compared to age-matched wild type controls (spines: 399.8 ± 33.1 particles/µm^2^; interneurons: 356.9 ± 26.2 particles/µm^2^) (Fig. 4A, B; Table 1). Thus, the average density of GluN1 was significantly decreased in the SLM by almost 2-folds in excitatory synapse on spines and interneurons in APP/PS1 mice (Mann–Whitney U test, ****P < 0.0001; Fig. 4I; Table 1).

**Table 1:** Number and density of immunoparticles for GluN1 at different excitatory synapses in the CA1 field, CA3 field and DG at 12 months of age

	CA1 SR		CA1 SLM		CA3 MF	CA3 A/C	DG (PP)
**WT**	Spines	Interneurons	Spines	Interneurons			
**Area of IMP clusters (n)** **(PSD of excitatory synapse)**	54	45	34	49	29	15	15
Mean (± SEM) (µm)	0.033 ± 0.002	0.056 ± 0.005	0.033 ± 0.002	0.071 ± 0.005	0.053 ± 0.004	0.038 ± 0.014	0.044 ± 0.007
Median gold particles	11	16	14	19	27	12.5	17
Range	36 − 2	59 − 2	34 − 4	44 − 7	53 − 6	21 − 3	35 − 10
Particles (CV)	0.59	0.68	0.47	0.48	0.47	0.40	0.42
**Density gold particles (µm** ^**2**^ **)**							
Mean (± SEM)	399.82 ± 33.15	356.95 ± 26.24	463.75 ± 32.67	337.91 ± 23.34	522.99 ± 26.52	366.45 ± 57.09	518.66 ± 54.54
Median	336.70	378.00	387.10	335.70	512.12	363.58	513.64
Range	1313 − 104	707.7–38.40	911.9–140.6	776.6-76.23	810.60-232.16	809.76-111.62	1017.20-203.93
Density (CV)	0.60	0.49	0.41	0.48	0.27	0.53	0.38
**APP/PS1**							
**Area of IMP clusters (n)** **(PSD of excitatory synapse)**	53	44	48	37	31	15	15
Mean (± SEM) (µm)	0.03 ± 0.02	0.056 ± 0.004	0.036 ± 0.004	0.073 ± 0.010	0.055 ± 0.006	0.034 ± 0.009	0.043 ± 0.005
Median gold particles	12	16	7	8	20	14	5
Range	28 − 2	49 − 1	25 − 1	37 − 1	54 − 2	22 − 6	12 − 1
Particles (CV)	0.52	0.54	0.72	1.1	0.39	0.59	0.58
**Density gold particles (µm** ^**2**^ **)**							
Mean (± SEM)	451.06 ± 28.40	349.60 ± 21.90	237.86 ± 16.58	184.37 ± 20.54	422.10 ± 21.65	397.62 ± 50.68	133.13 ± 19.19
Median	425.90	349.7	236.80	148.30	412.00	363.35	132.44
Range	979.9-78.51	708.9–30.00	545.7-22.72	454.1–9.72	676.00-143.56	715.92-197.67	239.09–20.38
Density (CV)	0.45	0.41	0.48	0.67	0.29	0.46	0,50

A/C, associational/commissural; MF, mossy fibres; PSD, postsynaptic density; PP, perforant pathway; SR, *stratum radiatum*; SLM, *stratum lacunosum-moleculare*

The area of IMP clusters established on spines and interneurons in the two layers of the CA1 field revealed no significant differences between wild type and APP/PS1 mice, suggesting no alteration in the synaptic size in APP/PS1 mice (Mann-Whitney *U* test, *P* > 0.1) (Table 1). In addition, the number of GluN1 immunoparticles per PSD and the average density of GluN1 immunoparticles per PSD in those synapses were quite variable (Table 1).

### Differential alteration of synaptic GluN1 in the CA3 field of APP/PS1 mice

**Fig. 5:**
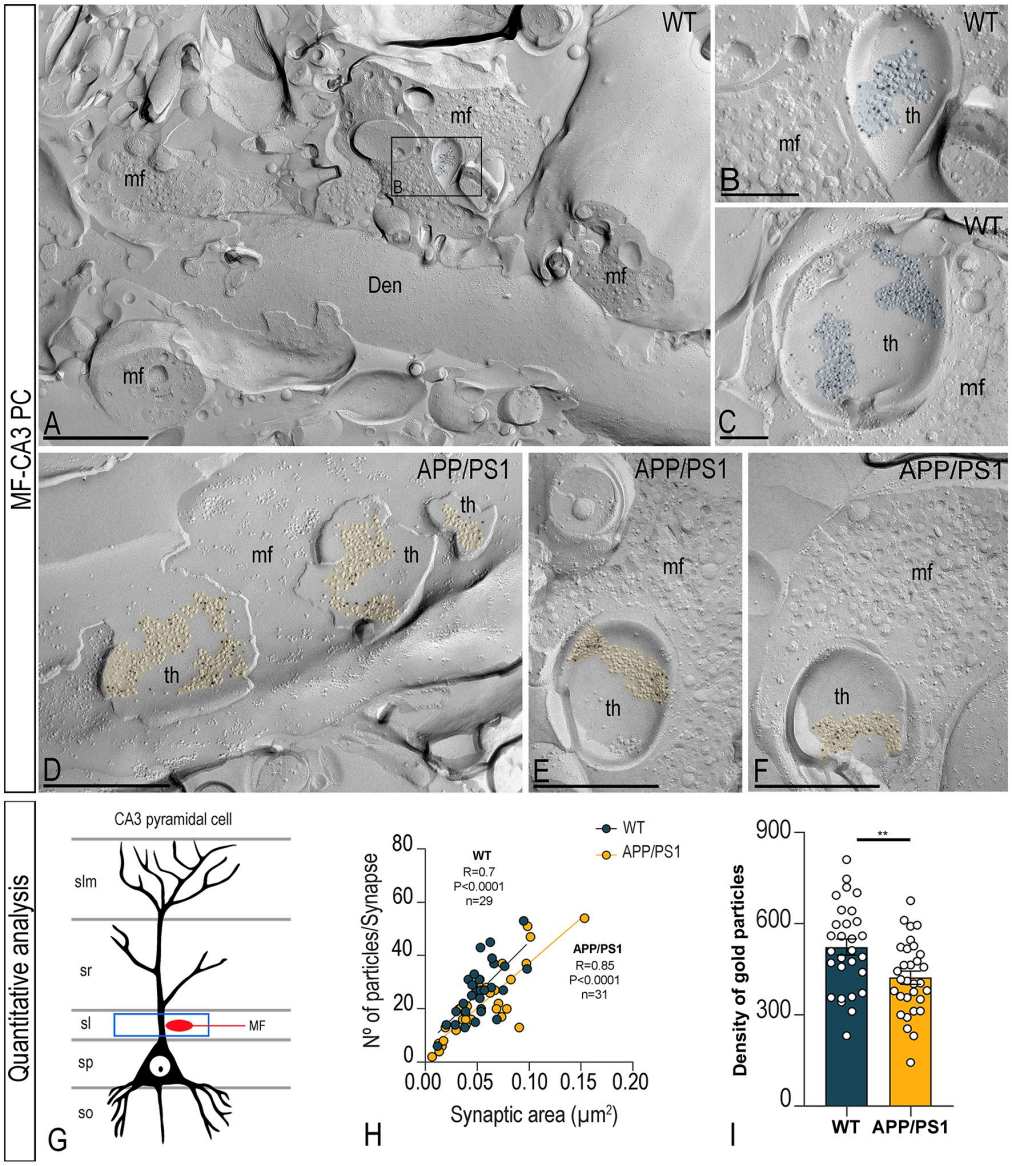
Reduced density of synaptic NMDARs in CA3 pyramidal cell-mossy fibre synapses of APP/PS1 mice. Electron micrographs of thorny excrescence (th) of pyramidal cells making excitatory synapses with mossy fibre terminals (mf) in the *stratum lucidum* of the CA3 field immunolabelled for GluN1, as detected using the SDS-FRL technique in wild type and APP/PS1 mice at 12 months of age. (**A-F**) Panel **A** shows a low-magnification image of the P-face of a thick dendrite (Den) of a CA3 pyramidal cell receiving several mf terminals in wild type mice. Electron micrograph in panel **B** shows a high-magnification image of the boxed area shown in panel **A**. Immunoparticles for GluN1 were distributed on thorny excrescence (th) of pyramidal cells making excitatory synapses with mf terminals in wild type and APP/PS1 mice. Scale bars: A,D, E,F, 500 nm; B,C, 200 nm. (**G**) Drawing of a CA3 pyramidal cell, with the blue box delineating the area used for the quantitative analysis in the *stratum lucidum* (sl). (**H**) Scatterplots of the number of immunoparticles for GluN1 versus size of excitatory synapses in the *stratum lucidum* in both wild type and APP/PS1 mice. There is a strong positive linear correlation between immunoparticle number and synaptic size (Pearson’s correlation test). (**I**) Mean densities of GluN1 in excitatory synapses in thorny excrescences in the hippocampal CA3 field in wild type and APP/PS1 mice. A significant reduction in the density of NMDAR immunoparticles were detected in APP/PS1 mice compared to age matched wild type (*n* = 3 animals per genotype; unpaired t-test, **P < 0.01)

The next analysis focused on unravelling the possible alteration of synaptic GluN1 in two excitatory synapses of the CA3 field. MF terminals are large and irregular in shape, packed with many synaptic vesicles and they establish multiple synapses with thorny excrescences of CA3 pyramidal cells (CA3 PC) in the *stratum lucidum* [31]. In SDS-FRL samples, MF were identified by ultrastructural criteria like wide membrane face and cross-fractured face containing numerous synaptic vesicles (Fig. 5A).

In both wild type and APP/PS1 mice, the majority of immunoparticles for GluN1 were randomly distributed over the entire PSDs with no apparent clustering but detected at a lower frequency in APP/PS1 mice (Fig. 5A-F). As expected from the variability in PSD areas analysed, the number of GluN1 immunoparticles per PSD in MF-CA3 synapses in both wild type and APP/PS1 mice was quite variable (Table 1). In contrast, the average density of GluN1 immunoparticles per PSD was less variable (Table 1). MF-CA3 PC synapses exhibited a strong positive correlation between the number of GluN1 immunoparticles and the area of PSDs in both wild type and APP/PS1 mice, consistent with the possibility that the number of NMDARs in individual synapses depends on the size of the MF-CA3 PC synapses (Fig. 5H). The area of the PSDs was similar in MF-CA3 PC synapses in wild type and APP/PS1 (Table 1). The mean number of immunoparticles for GluN1 in the MF-CA3 PC synapse was significantly reduced in APP/PS1 mice (422.10 ± 21.65 particles/µm^2^) compared to wild type mice (522.9 ± 26.5 particles/µm^2^) (unpaired t-test, **P < 0.01; Fig. 5I; Table 1). Thus, the average density of GluN1 was significantly decreased by 1.2-fold in the MF-CA3 PC synapse in APP/PS1 mice.

**Fig. 6:**
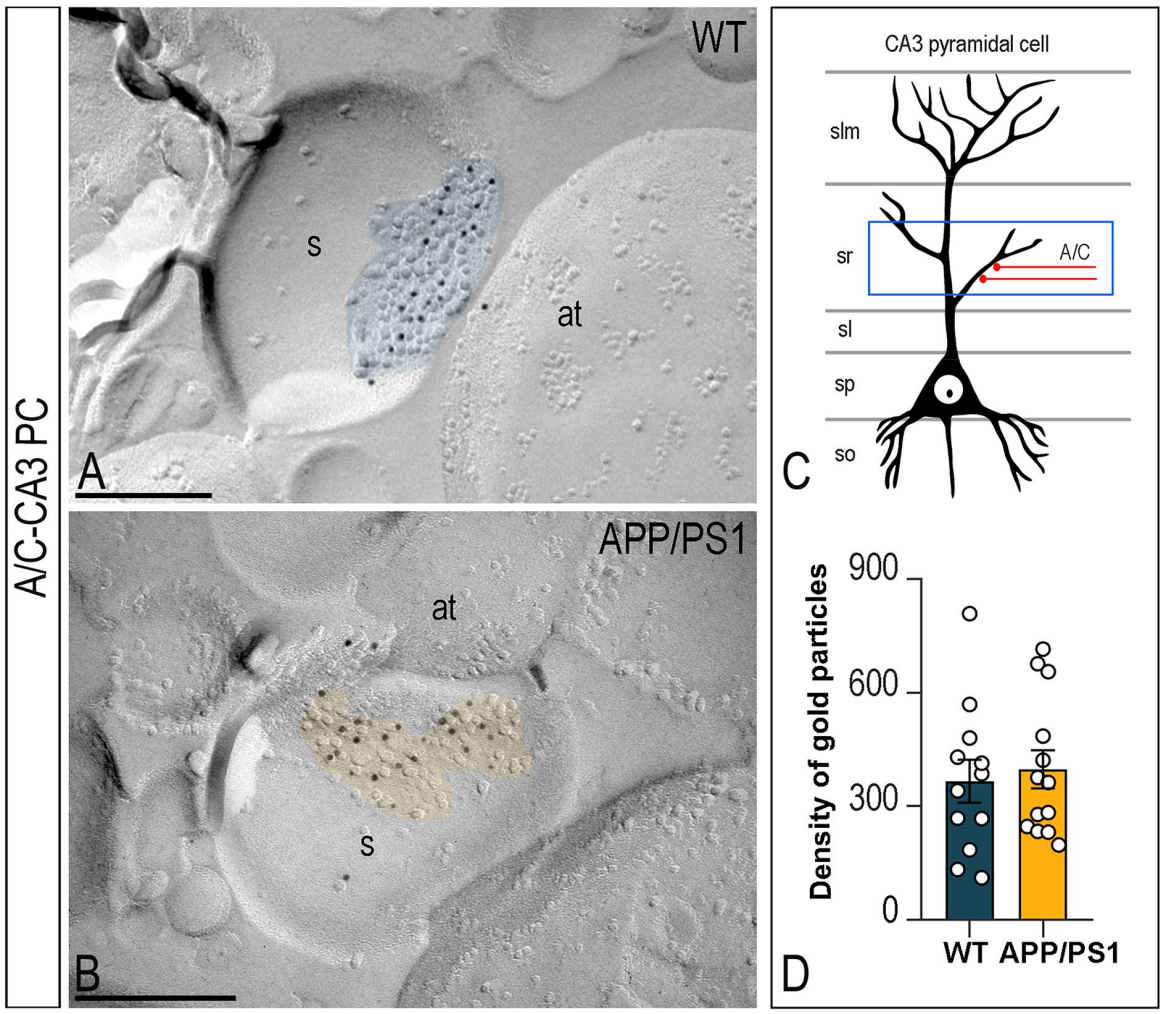
Density of synaptic NMDARs in CA3 pyramidal cell-A/C synapses of APP/PS1 mice. (**A**,** B**) Electron micrographs of pyramidal cell spines making excitatory synapses with associational/commissural (A/C) fibres in the *stratum radiatum* of the CA3 field immunolabelled for GluN1, as detected using the SDS-FRL technique in wild type and APP/PS1 mice at 12 months of age. Strong immunoreactivity for GluN1 was detected in the postsynaptic membrane specialisations (coloured with transparency in blue for wild type and in yellow for APP/PS1) in both wild type and APP/PS1 mice. Scale bars: A,B, 200 nm. (**C**) Schematic drawing of a CA3 pyramidal cell, with the blue box delineating the area used for the quantitative analysis in the *stratum radiatum* (sr). (**D**) Mean densities of GluN1 in A/C-CA3 PC synapses in wild type and APP/PS1 mice. No significant differences were observed (*n* = 3 animals per genotype; Mann-Whitney U test, *P* > 0.05)

CA3 PCs also receive recurrent CA3 collaterals A/C fibres. In both wild type and APP/PS1 mice, the majority of immunoparticles for GluN1 in A/C-CA3 PC synapses were distributed over the entire PSD with no apparent clustering (Fig. 6A, B). No difference in the density of GluN1 in A/C-CA3 PC synapses between APP/PS1 (397.6 ± 50.7 particles/µm^2^) and wild type mice (366.4 ± 57.1 particles/µm^2^) (*P* > 0.05; Fig. 6C, D; Table 1) was revealed.

### Altered number and density of GluN1 at DG perforant path synapses in APP/PS1 mice

**Fig. 7:**
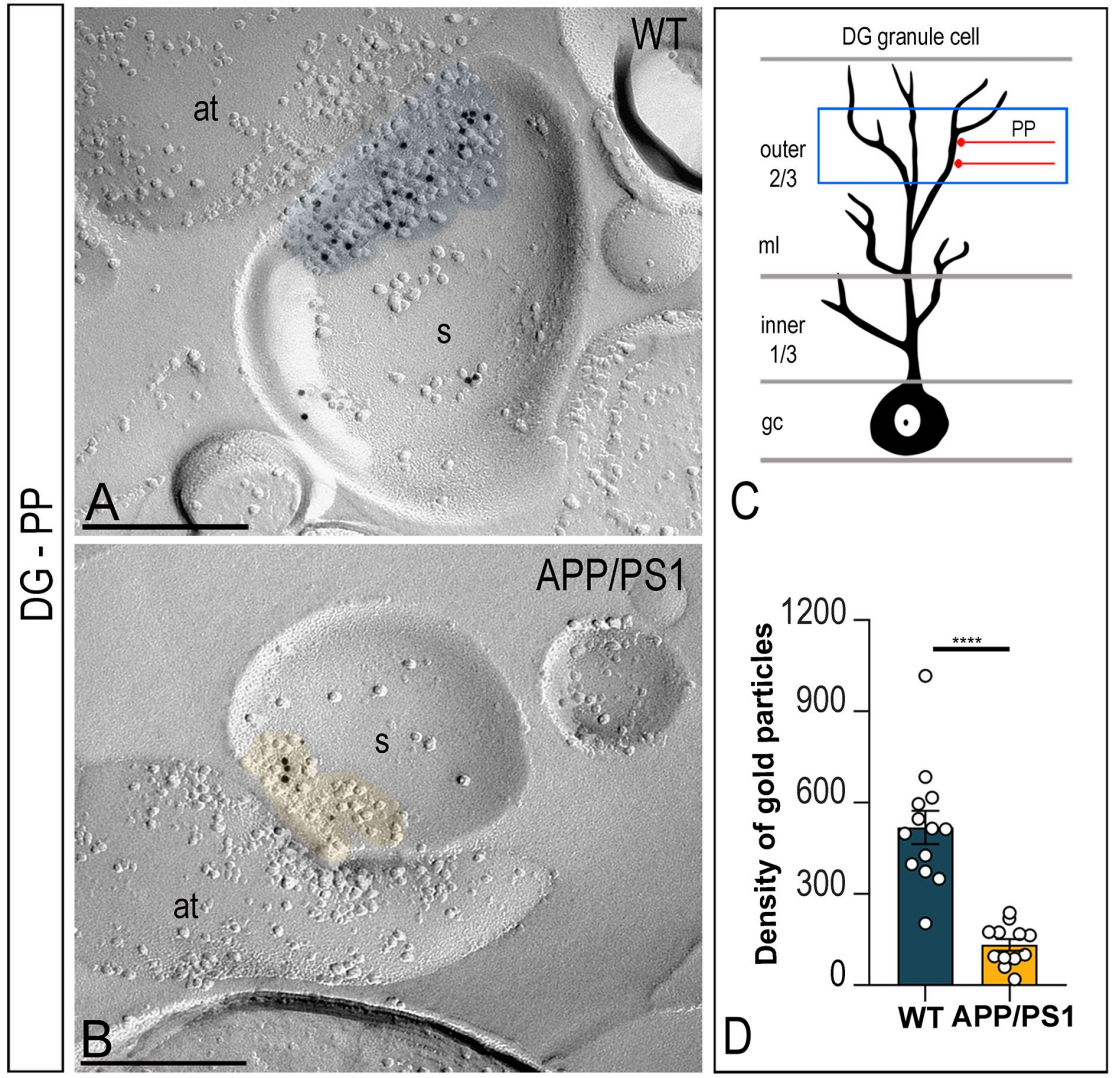
Reduced density of synaptic NMDARs in DG granule cells-perforant path synapses of APP/PS1 mice . (**A-B**) Electron micrographs of the DG showing immunoparticles for GluN1 at excitatory synaptic sites of spines of granule cells in the outer two thirds of the molecular layer, as detected using the SDS-FRL technique in wild type and APP/PS1 mice at 12 months of age. Postsynaptic membrane specialisations (IMP clusters, pseudo coloured with transparency in blue for wild type and in yellow for APP/PS1) show strong immunoreactivity for GluN1 in wild type, but weaker immunoreactivity in the APP/PS1. Scale bars: A,B, 200 nm. (**C**) Schematic drawing of a DG granule cell, with the blue box delineating the area used for the quantitative analysis in the outer two thirds of the molecular layer, where perforant path (PP) synapses are established. (**D**) Mean densities of GluN1 in DG-perforant path synapses in wild type and APP/PS1 mice. A significant reduction in the density of GluN1 immunoparticles were detected in APP/PS1 mice compared to age matched wild type (*n* = 3 animals per genotype; Mann-Whitney U test, ****P < 0.0001)

Possible changes in synaptic GluN1 in the perforant pathway of the DG were next analysed. Applying the SDS-FRL technique to samples from wild type and APP/PS1 mice, the immunoparticles for GluN1 were randomly distributed over the PSDs with no apparent clustering (Fig. 7A). However, less gold labelling was observed in APP/PS1 mice (Fig. 7B). The average area of PSDs was very variable and thus the number of GluN1 immunoparticles per PSD in both wild type and APP/PS1 mice was also quite variable (Table 1), in addition to the average density of GluN1 immunoparticles per PSD (Table 1) in DG perforant path synapses. Quantitative analyses revealed a significant reduction in the density of immunoparticles for GluN1 in DG perforant path synapses in APP/PS1 mice (133.1 ± 19.2 particles/µm^2^) compared to wild type (518.7 ± 54.5 particles/µm^2^). Thus, the average density of GluN1 was significantly decreased by 3.9-fold in the DG perforant path synapses in APP/PS1 mice (Mann-Whitney U test, ****P < 0.0001; Fig. 7C, D; Table 1).

## Discussion

Synaptic dysfunction and neuronal loss are well documented in AD and are major contributors to the neurodegeneration [20, 32, 33]. As a key component of the excitatory synaptic machinery, growing evidence implicates dysregulation of NMDARs as a major cause of cognitive impairments in AD and animal models of AD [34, 35]. This prompted us in this study to investigate possible alterations in the number and density of NMDARs at synaptic sites in Aβ pathology. We analysed the expression of the GluN1 subunit of NMDARs in all hippocampal subfields at 1-, 6- and 12-months-old APP/PS1 mice, and the synaptic organisation of GluN1 at 12-months-old APP/PS1 mice, a time when cognitive dysfunction is more severe in these animals [36]. The results of this study define the molecular organisation of GluN1 in different excitatory synapses of the hippocampus in physiological and pathological conditions and demonstrate for the first time a synapse-dependent reduction in the density of NMDARs in an amyloidogenic transgenic mouse model. Our data suggest that Aβ pathology selectively disrupts specific populations of excitatory synapses, leading to the progressive failure of their connectivity in the trisynaptic circuit of the hippocampus of our preclinical transgenic model.

The GluN1 subunit holds critical importance in the function of NMDARs, because of its ubiquitous presence as an obligatory subunit. As such, all NMDARs require the presence of GluN1 to be functional [1], and consequently a decrease in GluN1 expression would reduce functional NMDARs in a neuron. In the present study, our analysis showed that GluN1 expression in the hippocampus, cortex and caudate putamen was greatly affected by 12 months, but not detectable at earlier ages in the APP/PS1 mouse model. A similar age-dependent expression has been recently described in a model of tauopathy [21]. In physiological conditions, the hippocampus is the region with the expression levels of GluN1 among the highest in the brain [7], and consistent with these reports we found that GluN1 labelling was particularly strong in dendritic layers of the CA1, CA3 and DG subfields. In pathological conditions, previous studies have addressed mRNA and protein levels of NMDARs in AD brains. Furthermore, a downregulation of GluN1 in various stages of the disease has also been reported [28, 37–39]. Conversely, other studies have evidenced that GluN1 levels remain unchanged [40] or increased [41] in AD patient’s brains. Those studies were performed on homogenates prepared from the whole hippocampus, thus not allowing any detailed examination of different hippocampal subfields, which are known to be selectively vulnerable to degeneration in AD. Here, we took advantage of the histoblot technique [27] to establish the expression of GluN1 in all subfield and dendritic layers of the hippocampus in normal and pathological conditions. Our work in APP/PS1 mice showed a decline in the expression pattern of GluN1 in all subfields of the hippocampus and in a layer-dependent manner. This decline also took place in an age-dependent manner, with no changes at 1 and 6 months of age. Consistent with these findings, no alteration of NMDARs has been detected in the CA3 field at 6 months [42] or in the expression of GluN1 in the CA1 field at 8 months [43]. Interestingly, in agreement with our findings, western blots studies reported a marked decrease in the expression of GluN1 in the hippocampus in AD brains, but no changes in the expression of GluN1 in early stages of AD, suggesting that the decrease in expression takes place in an advance stage [44].

During aging, the hippocampus shows a decrease in volume [45], pyramidal cells show a decrease excitability and altered synaptic plasticity [46], and NMDARs become hypofunctional [47], which correlates with a decline in learning and memory in elderly. In this context, it has been shown that Aβ oligomers interact with GluN1 in a transgenic model of AD [48]. Furthermore, administration of Aβ oligomers to organotypic slices containing pyramidal neurons decreased dendritic spine density and reduced NMDAR-mediated Ca^2+^ influx [49], with the NMDAR antagonist memantine reversing this loss [50]. The possible pathogenic alteration in the number and density of NMDARs at synaptic sites has not yet been determined in APP/PS1 mice. We analysed the organisation of GluN1 in different populations of excitatory synapses in the CA1 and CA3 fields and DG. The glutamatergic pathways established between these three subfields make up the trisynaptic circuit formed by the perforant pathway to granule cell synapse in the DG, the MF projection from DG granule cells to CA3 pyramidal cells, which in turn project to CA1 pyramidal neurons via the Schaffer collateral pathway [51]. This circuit allows the signal entering the hippocampus to return to the cortical areas from which it originated.

In the CA1 field, pyramidal cells and interneurons receive most of their excitatory inputs from the perforant path, which is originated in the entorhinal cortex (EC). It then travels through the *stratum lacunosum-moleculare* and Schaffer collaterals and commissural fibres which arises from the ipsilateral and contralateral CA3 PCs residing in the *stratum radiatum* [51]. Both pyramidal cells and interneurons express high levels of mRNA and protein for GluN1 [8, 9]. Its synaptic organisation in both neuron populations has been previously studied using the post-embedding technique [13–15]. However, the high labelling efficiency of the SDS-FRL technique , for NMDARs compared with the conventional post-embedding method provided us with a powerful tool to quantify with a nanoscale spatial resolution in any excitatory synapse [52, 53]. Our measurements confirmed that the number of NMDARs in spine and interneuron synapses correlated with synaptic area in normal conditions and Aβ pathology, similarly to data described in P301S mice [21]. Furthermore, given that morphological parameters of postsynaptic membrane specialisation play a critical role in synaptic transmission [54], the possibility that the decrease of synaptic GluN1 in any subfield is accompanied by PSD changes in Aβ pathology was rejected by our observations of similar synaptic sizes in both control and APP/PS1 mice.

In AD, the CA1 field is one of the most influenced and altered regions [55]. It is involved in spatial orientation, learning, and different aspects of memory, such as consolidation and retrieval [56]. The impairment of these functions is related to the core clinical symptom in AD patients [55] and NMDARs are likely to be implicated, although a key issue is to unravel how this receptor is altered in Aβ pathology. Interestingly, we report here that the density of GluN1 in spine synapses is significantly reduced in the *stratum lacunosum-moleculare* but unaltered in the *stratum radiatum* in APP/PS1 mice, suggesting changes in a projection-dependent manner. Following previous studies reporting alterations in GABAergic activity in models of amyloidosis [57], we investigated potential disruption of NMDARs in interneurons. Our findings indicate that interneurons show the same layer dependent GluN1 alteration as for the dendritic spines. This suggests the existence of disrupted GABAergic transmission in APP/PS1 mice, which is in agreement with studies showing increased activity in the CA1 field in APP/PS1 mice [58] and in MCI patients [59]. Previous work of our group has demonstrated that AMPARs were reduced in the *stratum radiatum* in the same amyloidogenic transgenic mouse model [60], suggesting the existence of an imbalance in the NMDAR/AMPAR ratio in Schaffer collateral synapses.

In the CA3 field, two main types of glutamatergic inputs of CA3 PCs are MF and A/C fibres. They are segregated along the surface of CA3 PCs and can be distinguished based on their structural features and functional properties in episodic memory encoding and recall [61]. Our findings showing the synaptic organisation of GluN1 at hippocampal MF and A/C synapses are consistent with previous observations using immunoelectron microscopy [9, 62]. The present data are also compatible with findings reporting that MF LTP is dependent on postsynaptic NMDARs [18, 63], and that they regulate the excitability of the CA3 PC recurrent network [64]. Existing evidence from a mouse model of AD-related Aβ accumulation supports a functional deficit in MF synapses [42, 65]. Consistent with these functional data we found that synaptic NMDARs at MF-CA3 PC synapses were reduced in the APP/PS1 transgenic mice. However, applying the same methodological approach we did not detect any alteration in A/C-CA3 PC synapses. The two CA3 synapses differ in their functional properties. The A/C synapses are thought to be essential for short-term memory, whereas MF synapses are required for the acquisition of contextual memories [66]. Therefore, Aβ pathology disrupt synaptic NMDARs in a synapse-dependent manner in the same neuron population in the CA3 field. This differential alteration seems to be age-dependent, as previous functional studies did not detect changes in APP/PS1 mice of 6 months of age [42]. Finally, GluN1 has also been detected at presynaptic sites at MF-CA3 PC synapses [62]. However, our analysis focused exclusively on postsynaptic NMDARs, so we cannot exclude the possibility that presynaptic receptors are also altered in APP/PS1 mice. Future studies are needed to unravel this issue.

The DG is involved in episodic and spatial memory and the exploration of novel environments [67]. The plasticity of synaptic transmission within the DG, the main gateway for EC inputs to the hippocampus, play a critical role in the processing of cortical information [67]. Layer II neurons of the EC project to the outer two-thirds of the DG molecular layer via the perforant path [68]. This is vulnerable in AD pathogenesis due to the loss of excitatory synapses [69], reduction in the expression of synaptic proteins [70], dramatic loss of layer II entorhinal neurons [71] and the presence of amyloid plaques and neurofibrillary tangles. In this study, we have delineated the synaptic pathology of the outer two-thirds of the molecular layer in by revealing the disruption of GluN1 at DG-PP synapses. Consistent with these findings, Aβ reduces the surface expression of NMDARs in granule cells of the DG [72]. In addition, DG neurons require intact NMDAR function for survival in aged mice [73]. Therefore, the large synaptic reduction of GluN1 may be a critical parameter involved in the neurodegeneration of neurons in the DG.

Although our data provide novel insight into the alteration of NMDARs in AD pathology, we must acknowledge some caveats of the study pointing that molecular diversity of these receptors impacts neuronal function. First, the GluN2A-D subunits enhance the activity of NMDARs when associated with the GluN1 subunit, conferring different agonist/antagonist affinities to the GluN1/GluN2 heteromeric receptors and producing different gating behaviours and responses to Mg^2+^ [74–76]. Our study did not target GluN2 subunits due to the lack of antibodies working efficiently for SDS-FRL. Second, there are eight different splice variants of the mRNA for the GluN1 subunit that exist in the brain showing variation in regional profiles [77] and the anti-GluN1 antibody used here recognised all of them. Therefore, the contribution of specific GluN1 splice variants in AD pathology could not be determined. Lastly, GluN3 subunits co-assemble with GluN1 and GluN2 subunits to form triheteromers with biophysical properties distinct from those of GluN1/GluN2 diheteromers [78]. However, no antibodies against GluN3A or GluN3B have been validated for SDS-FRL.

In summary, this study is the first to report a differential synaptic decline of NMDARs in the trisynaptic circuit of the hippocampus in Aβ pathology. GluN1 was significantly decreased by almost 4-fold in the DG perforant path synapses, by almost 2-folds in CA1 perforant path synapses on spines and interneurons and by 1.2-fold in the MF-CA3 PC synapses, but unaltered in A/C-CA3 PC synapses and CA1 Schafer collateral synapses. Therefore, hippocampal synapses are not equally affected by Aβ pathology in the trisynaptic circuit. This differential disruption of synaptic GluN1 indicates functional changes in the subfields of the hippocampus, which may have a significant impact on cognitive function in APP/PS1 mice. This data provides mechanistic insights to understand how glutamate receptor changes in AD, a key information that may lead to new therapeutic approaches to target specific components of the glutamatergic signalling pathway.

## Data Availability

All data used and/or analysed during the current study are available from the corresponding author on reasonable request.
